# POETRY AND UNCOVERING THE MYSTERY OF SOCIAL PROBLEMS: AN ARTS-BASED INQUIRY TO AGING CARE

**DOI:** 10.1093/geroni/igac059.2139

**Published:** 2022-12-20

**Authors:** Heng Wu, Peter Szto

**Affiliations:** The University of Nebraska at Omaha, Omaha, Nebraska, United States; The University of Nebraska at Omaha, Omaha, Nebraska, United States

## Abstract

This research focuses on a current social welfare problem: unemployment and the loss of employer-based health insurance among non-elderly adults aged 18 to 64. The literature covers this social problem in terms of health status, access to medical care, employed versus unemployed working-age adults, mitigating the risks of unemployment, and loss of health insurance via the Consolidated Omnibus Budget Reconciliation Act of 1985 (COBRA) and the Health Insurance Portability and Accountability Act of 1996 (HIPAA). As good as the findings are, they are methodologically limited by looking at only half the story. This research asks a different question: how might the humanities and arts-based perspective address the social problem? It aims at answering why the employment status is related to health and health insurance. Chinese poetry has a long rich tradition of expressing insights about the inner life since antiquity to the present. These reflections include practice wisdom and keen observations on aging. This poster presents case findings on the use of Chinese poetics to inform aging care in the West. Each case provides discussions on why social welfare system comes up short to solve the problem and how the social welfare system can be effectively changed. Researchers use survey data to compliment Chinese poetic insights about employment status and insurance status to illustrate the correlation between respondents’ health status with versus without employment. Findings are hoped to interpret the role of family as a social unit where it consists in social welfare along with the speculative inquiry of poetry.

